# Electrical Control of Valley Polarized Charged Exciton Species in Monolayer WS_2_

**DOI:** 10.1021/acsnano.4c11080

**Published:** 2024-10-22

**Authors:** Sarthak Das, Ding Huang, Ivan A. Verzhbitskiy, Zi-En Ooi, Chit Siong Lau, Rainer Lee, Calvin Pei Yu Wong, Kuan Eng Johnson Goh

**Affiliations:** † Institute of Materials Research and Engineering (IMRE), Agency for Science, Technology and Research (A*STAR), 2 Fusionopolis Way, Innovis #08-03, Singapore 138634, Singapore; ‡ Quantum Innovation Centre (Q.InC), Agency for Science Technology and Research (A*STAR), 2 Fusionopolis Way, Innovis #08-03, Singapore 138634, Singapore; § Science, Mathematics and Technology, Singapore University of Technology and Design, 8 Somapah Road, Singapore 487372, Singapore; ∥ Department of Physics, National University of Singapore, 2 Science Drive 3, Singapore 117551, Singapore; ⊥ Division of Physics and Applied Physics, School of Physical and Mathematical Sciences, Nanyang Technological University, 50 Nanyang Avenue, Singapore 639798, Singapore

**Keywords:** valley polarization, trion and quinton, resonant excitation, motional narrowing, exchange interaction, resonant coupling

## Abstract

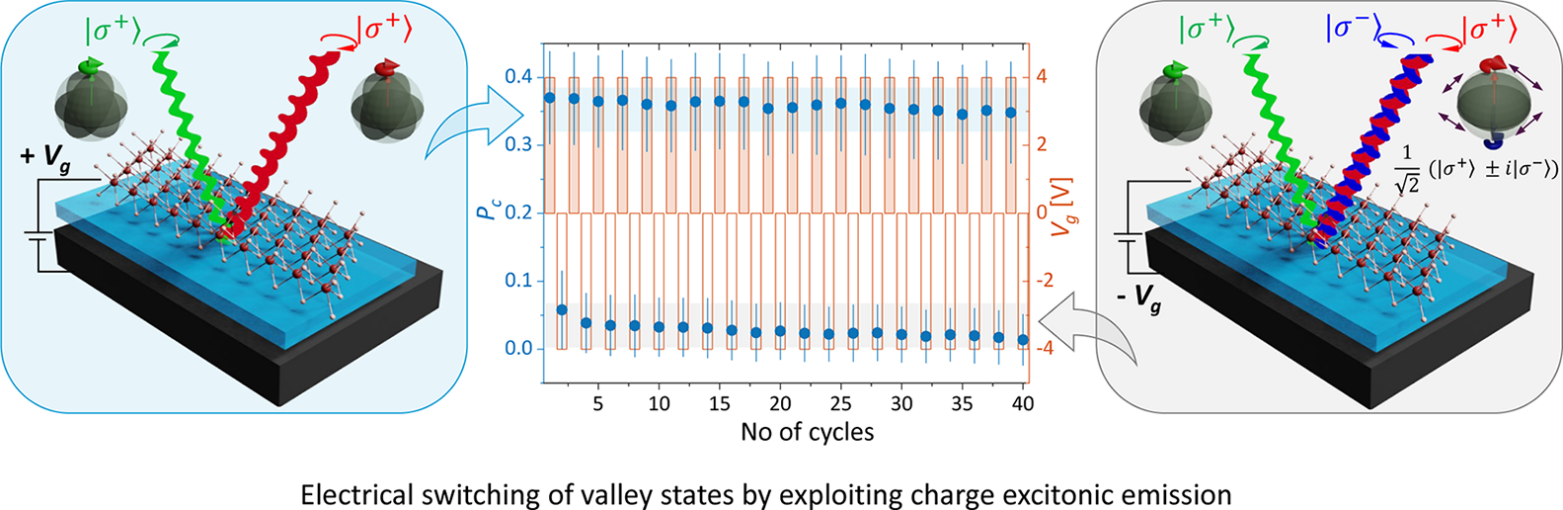

Excitons are key to the optoelectronic applications of van der Waals semiconductors, with the potential for versatile on-demand tuning of properties. Yet, their electrical manipulation remains challenging due to inherent charge neutrality and the additional loss channels induced by electrical doping. We demonstrate the dynamic electrical control of valley polarization in charged excitonic states of monolayer tungsten disulfide, achieving up to a 6-fold increase in the degree of circular polarization under off-resonant excitation. In contrast to the weak direct tuning of excitons typically observed using electrical gating, the charged exciton photoluminescence remains stable, even with increased scattering from electron doping. By exciting at the exciton resonances, we observed the reproducible nonmonotonic switching of the charged state population as the electron doping is varied under gate bias, indicating a resonant interplay between neutral and charged exciton states.

## Introduction

Excitons in monolayer transition metal dichalcogenides (TMDs) have high binding energy,^1−3^ enabling strong resonances even at room temperature. This makes TMDs highly attractive for a wide range of applications in optoelectronics^4−7^ and quantum technologies.^8−10^ Particularly, the valley-dependent optical selection rules in monolayer TMDs, due to the presence of two inequivalent valleys with spin–valley locking and a direct bandgap, allow high degree of optical control over valley polarizations^4,11−15^ and valley coherence.^16,17^

Although neutral excitons can host highly polarized optical states,^11,13−17^ their charge neutrality makes excitons insensitive to electrical bias, limiting the on-demand control of excitonic properties, such as the resonance energies, radiative and nonradiative lifetimes, and photoluminescence (PL) brightness. Moreover, these states are susceptible to environmental noise, which can result in a loss of quantum information. The challenges are further exacerbated by interactions with charges, spins, disorders, and thermal excitations. As a result, the need for an electrically controlled, valley-polarized photon source remains unfulfilled.

Earlier studies have shown that the modulation of valley polarization of neutral and charged states can be implemented with gate voltage,^18,19^ temperature,^19^ screening,^20,21^ strain^22^ or magnetic field.^23^ However, achieving simultaneous electrical control while maintaining a high yield of polarized photons remains a challenge. Additionally, most of the methods used to achieve external control with valley-polarized excitons lead to a compromise in photon yield due to various nonradiative losses.

On the other hand, the monolayer TMDs can host a nexus of many-body quasiparticles such as three-particle charged excitons or trions,^24−29^ four-particle biexciton,^30−32^ five-particle charged biexcitons or quintons^33−41^ and even higher many body species.^42,43^ Typically, the formation of charge excitons is enabled by finite doping, where optically generated neutral excitons capture free electrons or holes. Beyond the generation of excitonic quasiparticles, the interactions between excitons are also influenced by carrier density, screening, and system temperature.^44,45^ This can lead to bandgap renormalization,^46^ exchange interaction,^47^ and Columbic screening,^20,21^ impacting optical transport, radiative lifetime, and leading to Auger recombination^48^ at higher carrier densities. Additionally, excitons can interact with trapped charges to form defect-bound excitonic states or localized trions, which can be activated by optical pumping or electrostatic gating at low temperatures.^49,50^ The experimental observation of these complex states stems from the high binding energy of the underlying excitons, offering potential mechanisms for electrical control of excitons via charged species. To overcome the limitations of neutral exciton and explore the valley-polarized charge states, we utilize optically generated valley-polarized emission from the charged states, controlled via electrical injection.

While these charged exciton species are electrically controllable, preserving high valley polarization, a crucial requirement for valleytronic applications, has not yet been demonstrated. In this paper, we demonstrate that valley polarization originating from the charged exciton species can be electrically switched between high- and low-polarized states along with its dynamic modulation. This work shows that valley polarization can be manipulated using only electrical bias under nonresonant excitation. The study emphasizes the reproducible and stable electrical switching of the valley polarization in monolayer using charged states, offering several advantages including higher photon yield and spectral tunability. The study suggests that electrically controlled valley-polarized emission from charged states could overcome the traditional limitations of neutral excitons and marks a significant step toward achieving practical applications of valleytronic devices. We discuss the implications of these findings in the context of resonant control of charged excitons, where the charged exciton population can be controlled with neutral exciton resonance while retaining valley polarization. Our findings not only provide insight into how valley-polarized charge states can be manipulated in monolayer TMDs but also emphasize their potential as reliable platforms for preserving quantum information in the presence of environmental disturbances or even when there are charge defects in the samples.

## Results and Discussions

**Figure 1:**
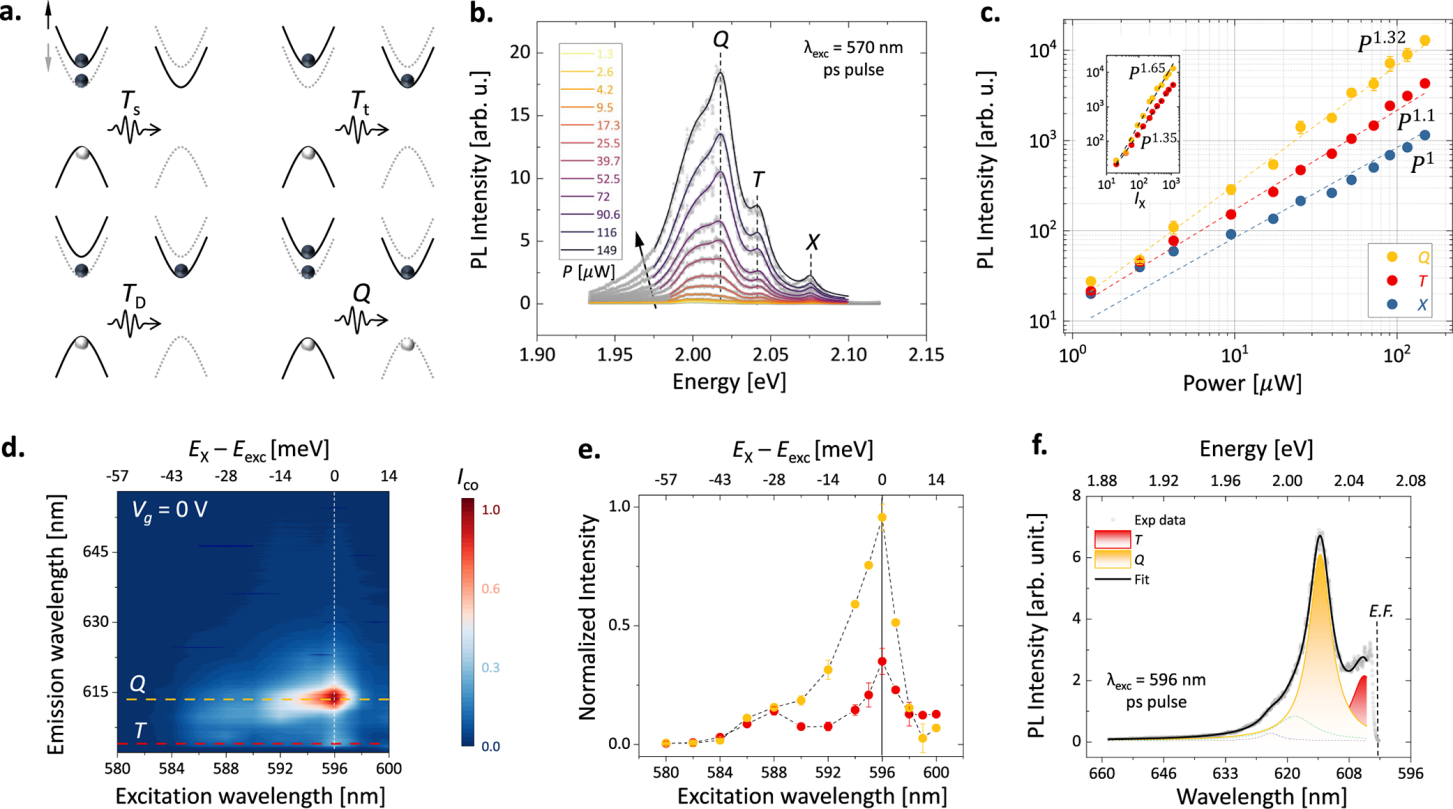
PL measurements of the excitonic complexes for resonant and nonresonant excitations. (a) The spin-valley configurations of *e* – *h* pair for intravalley (singlet trion, *T*_*s*_), intervalley (triplet trion, *T*_*t*_), dark trion (*T*_*D*_) and the combination of bright exciton (*X*) and *T*_*D*_ or the five-particle charged biexcitons (quintons, *Q*). The solid (dotted) line represents up(−down) spin bands at the zone edges (*K* and *K*^′^ valleys). (b) The power (*P*) dependent PL intensity (black arrow refers to increasing power) of monolayer WS_2_ sandwiched between two *h*BN layers for nonresonant λ_*exc*_ = 570 nm ps pulsed excitation at *T* = 4.6 K. The spectra consist of three main peaks, which have been identified through Lorentzian peak fittings based on their energy position and power law analysis. The raw data is represented by solid symbols, while the cumulative fit is shown with solid lines. The energies of the prominent peaks are identified as exciton (*X* at 596 nm or ∼2.078 eV), trion (*T* at 606 nm or ∼2.044 eV) and charged biexciton or quintons (*Q* at 614 nm or ∼2.019 eV). The vertical dashed lines represent the individual peak positions at different power of excitations. (c) The log–log plot for the power law analysis where the peak intensities vs power are fitted as *P* ∝ *I^α^*. The *Q* peak intensity grows super linearly against the *P* with a coefficient of *α*_*Q*_ = 1.32 ± 0.04, while for *T* the *α*_*T*_ = 1.1 ± 0.04. Inset shows the charged exciton intensity fitted against neutral exciton intensity (*I*_*X*_) where the *α*_*Q*_ = 1.65, following a super linear trend. (d) The normalized intensity map of the excitation wavelength-dependent emission spectra recorded below the exciton energy at *T* = 4.6 K. The top axis represents the excess energy [energy difference between laser excitation (*E*_*exc*_) and neutral exciton (*E*_*X*_)]. The red (yellow) horizontal dashed line denotes the *T* (*Q*) energy resonance across the excitation. The brightest region corresponds to the exciton resonance (λ_*exc*_ = 596 nm) denoted by the white vertical dashed line with *V*_*g*_ = 0 V. (e) The normalized intensity of the charged states (following the horizontal lines in the previous figure) over the photoluminescence excitation (PLE) wavelengths. The intensity is at its maximum when the excess energy is at its minimum. The black dashed lines serve as a guide to the eye of the exciton resonance. (f) Representative microphotoluminescence (μPL) spectra of the monolayer for resonant λ_*exc*_ = 596 nm excitation (following in vertical line cut from figure d). The solid vertical line denotes the exciton resonance (excitation wavelength, λ_*exc*_ = 596 nm) and the dashed line denotes the cutoff filter for reference. The individual peaks are fitted with Lorentzian multipeak fitting to show the *T* and *Q* energy states with an incident laser power of ∼8.7 μW.

In the presence of free carriers, an optically generated bright exciton (*X*) in monolayer tungsten disulfide (1L WS_2_) can capture an electron, forming intra- or intervalley trions (singlet *T*_*s*_ or triplet *T*_*t*_ trions) while a dark exciton can form dark trion states (*T*_*D*_).^28,29,51,52^ Additionally, due to the presence of the lowest energy dark states in WS_2_, the bright exciton can combine with *T*_*D*_ to form a five-particle quinton (or charged biexciton state, *Q*).^35,36,48^ We are particularly interested in the multiparticle *Q* state because of its higher photon yield, superlinear response,^40^ higher electrical tunability,^48^ and significant nonlinear optical properties.^41^ The different configurations of the energy states are schematically presented in Figure 1a.

In our sample (D1), 1L WS_2_ is sandwiched between two hexagonal Boron Nitride (*h*BN) layers. When we excite the sample with a picosecond (ps) pulse laser at 570 nm excitation and cryogenic temperature (*T* = 4.6 K), the microphotoluminescence (μPL) spectra predominantly feature three main peaks. The line width of the individual peaks is broader due to excitation with a ps-pulsed laser (see Methods for the details). Hence, we first analyzed the PL intensity dependence with power for each peak to confirm their origin. The power-dependent PL intensity from the sample is presented in Figure 1b, where all the individual spectra are fitted with multipeak Lorentzian curves (representative spectra fitting is shown in Supporting Information S1). We identify the peaks based on their energy position and the power-dependent PL, illustrated in the log–log plot in Figure 1c. The intensity (*I*) of the individual peaks is plotted against the excitation power (*P*) and fitted to extract the power coefficient (α). Based on the power-law analysis (where *I ∝ P*^*α*^), we attribute the three peaks to exciton (*X* at 596 nm or ∼2.078 eV), trion (*T* at 606 nm or ∼2.044 eV), and charged biexciton or quinton (*Q* at 614 nm or ∼2.019 eV). The low energy shoulder peak below the *Q* state is likely to be associated with the *T*_*D*_ state.^29,52^ The *Q* peak intensity exhibits a superlinear trend with *α*_*Q*_ = 1.32 ± 0.04, in accordance with previous reports.^29,33,35,36,52^ In the rest of the manuscript, we will primarily focus on the PL from this energy state and its modulation under electrical bias.

The exciton population primarily governs the population of the coupled charged excitonic quasi-particles. Further, the exciton population can be controlled efficiently by resonant (λ_*exc*_ = 596 nm) vs nonresonant excitation (λ_*exc*_ = 570 nm). Figure 1d shows a 2D color map of photoluminescence excitation (PLE) spectra measured across the neutral exciton resonance (∼596 nm, horizontal dashed line) at *V*_*g*_ = 0 V. At excitation energies higher than the neutral exciton emission, the spectra reflect an off-resonant regime where the PL emission of *T* and *Q* peaks is low due to fast nonradiative recombination processes.^40^ However, as the excitation energy approaches the exciton resonance (*E*_*X*_), PL peaks of the individual energy states get stronger. The peak intensity reaches the maximum at the neutral exciton resonance (596 nm at *V*_*g*_ = 0 V), then decreases due to sub-bandgap optical excitation. The red (yellow) horizontal dashed lines follow the *T* (*Q*) energy states. Figure 1e shows the normalized intensity profile at *T* and *Q* energy positions as a function of excitation energy, highlighting the PL enhancement of the charged excitons at the *X* resonance (shown by the black line) due to efficient down-conversion from the resonantly formed neutral excitons. This also denotes the efficient coupling between the neutral and charged states.^27,53^Figure 1f represents the vertical line cut from Figure 1d at exciton resonance with λ_*exc*_ = 596 nm. The solid vertical line denotes the excitation wavelength at 596 nm, while the dashed vertical line represents the edge of the cutoff filter for reference (see the Methods section for further details). The *T* and *Q* energy states and intensities below the *X* state are identified for resonant excitation at *X* using Lorentzian curve fittings.

**Figure 2:**
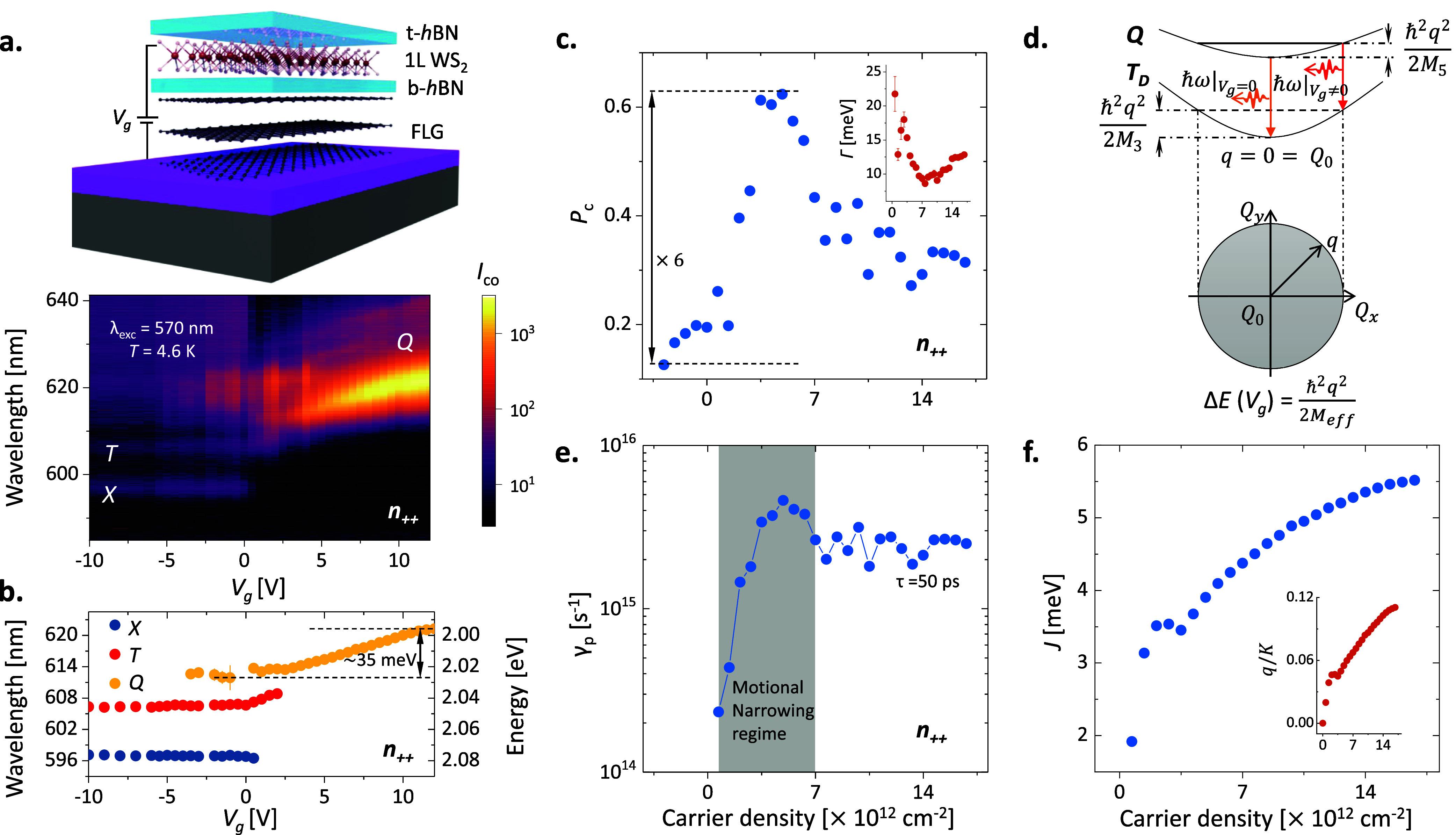
Gate-dependent spectroscopy and dynamic modulation of valley polarization for the charged biexcitons. (a) The schematic diagram in the top panel shows the device D1 where monolayer WS_2_ is sandwiched between two *h*BN layers (*t* and *b* refers to the top and bottom *h*BN layers) over the few-layer graphite (FLG), serving as a back gate (*V*_*g*_). The two-dimensional (2D) colorplot for the *V*_*g*_ dependent μPL spectroscopy under nonresonant excitation (λ_*exc*_ = 570 nm ps pulsed) shown in the bottom panel under copolarized mode (where *I*_*co*_ is the intensity under σ^+^ excitation and σ^+^ detection, σ^+^/σ^+^) of excitation. The energy states are mostly featureless for *V*_*g*_ < 0 V. However, when electron injection starts (for *V*_*g*_ > 0 V), the neutral state intensity drops and charged state intensity grows monotonically with a pronounced red shift dominated by the *Q* state in the high *n*-doped region (labeled as *n*_++_). Here, the intensity of the 2D map is presented on a logarithmic scale. (b) The gate-dependent variation in energy positions corresponding to different energy states, extracted from the individual peak fittings. The *Q* state shows a noticeable red shift of ∼35 meV compared to the neutral region under positive gate bias. (c) The gate-dependent valley polarization  or the degree of circular polarization (*P*_*c*_) of the *Q* state. The *P*_*c*_ shows nonmonotonic behavior as the carrier density increases. Inset: Corresponding line width (Γ) of the *Q* state extracted from the fitting across the carrier density. (d) Representative diagram describing about the *V*_*g*_ dependent transitions from charged biexcitonic state (quinton state, *Q*) to a *T*_*D*_ state. At *V*_*g*_ = 0 V, the vertical transition (represented by the orange arrow) is at *q* = 0 = *Q*_0_ while the transition occurs ( at *V*_*g*_ > 0 V) at higher *q* value (represented by the red arrow) for higher *V*_*g*_ because of Pauli blocking. In-plane projection of the same is presented in the bottom panel in the *Q*_*x*_ – *Q*_*y*_ plane for reference. (e) The extracted scattering rate, γ_*p*_, for a given lifetime of τ = 50 ps for *Q* state.^33,35,38,40^ The scattering rate increases initially along with the increase in carrier density and then saturates. The increment in *P*_*c*_ along with γ_*p*_ refers to the motional narrowing regime (shaded region). (f) The extracted exchange interaction (*J*^*LR*^(*q*)) variation with carrier density along with the normalized CoM momenta dispersion (*q/K*) mapped from the kinetic energy difference (inset) for the experimental red shift from Figure 2b using Equation 4. The *J*^*LR*^(*q*) increases with increasing carrier density.

The population of the energy states related to charged excitonic species can be externally controlled with electrical bias. To verify that the *h*BN-capped monolayer is placed on a few-layer graphite (FLG) serving as a back gate (*V*_*g*_). The schematic of the device D1 is shown in the top panel of Figure 2a. When an external DC bias is applied to the monolayer *via V*_*g*_, as we can observe in Figure 2a,b, the energy states exhibit a shift in energy position, change in intensity, and variation in the valley polarization as reported in other works.^35,40,48^ The two-dimensional colorplot for gate-dependent PL spectra for nonresonant excitation (λ_*exc*_ = 570 nm ps pulse) is presented in the lower panel of Figure 2a. The representative spectra and fittings are shown in Supporting Information S2.

Due to the optical selectivity and distinctive spin-valley configuration, the right (left) circularly polarized light can selectively address the *K*(*K*′) valley in 1L WS_2_. Here, the PL intensity is recorded for both the co-(σ^+^ excitation and σ^+^ detection, σ^+^/σ^+^) and cross-polarized (σ^+^ excitation and σ^–^ detection, σ^+^/σ^–^) modes (see Methods section for further details). The intensity mapping presented in Figure 2a is for the copolarized mode, while the cross-polarized spectra are shown in Supporting Information S3. The corresponding degree of circular polarization () in steady-state can be obtained from the relation:
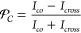
1where *I*_*co/cross*_ denotes the intensity of the co- or cross-polarized PL. All the energy states discussed above show a distinct polarization upon circularly polarized light excitation (the 2D color map of  as a function of applied *V*_*g*_ is shown in Supporting Information S2). The peak positions of the individual energy states are extracted from the individual peak fittings and plotted in Figure 2b. We observe three regions with distinct behaviors as follows:

(i) For *V*_*g*_ < 0, the individual energy state’s position and corresponding intensity remain unaltered. The charged excitonic complex states are highly sensitive to doping. However, due to the relative alignment between 1L WS_2_ and graphite contact, it is difficult to inject holes into the WS_2_.^54,55^ Hence, the exciton and other excitonic complexes undergo negligible modulation while *V*_*g*_ is negative.

(ii) At small positive *V*_*g*_, the electrons are injected into the system. These free electrons are captured by the optically created neutral excitons (*X*) and thus form three-particle negatively charged exciton (*T*) or five-particle quintons (*Q*, charged biexciton). Also, in this process, neutral excitons transfer their oscillator strength to the charged species. As a result, the intensities of the *T* and *Q* states start to increase (see Supporting Information S3) while the *X* intensity fades away.

(iii) As doping increases further (*V*_*g*_ > 0), the spectrum is dominated by the five-particle *Q* state. This is because the lowest energy state in 1L WS_2_ is dark,^56−58^ thus favoring the formation of *Q* states rather than *T* states. This *Q* state experiences monotonic enhancement of the intensity and gradual red-shift due to Pauli blocking with the increasing *V*_*g*_(36,48) (marked as *n*_++_ in the Figure 2a,b). The red shift of the *Q* state is almost linear over the experimental range, with a slope of ∼35 meV per decade.

The  corresponding to the *Q* state is presented in Figure 2c. As shown in Figure 2a,b, the energy states are not sensitive to the external bias for the *V*_*g*_ < 0 V region. This is also true for . As the intensity of the *Q* state grows with the number of free electrons, the  of the *Q* state is modified nonmonotonically. As shown in Figure 2c,  changes from <10% to >60% at carrier density (*n*) of ∼4 × 10^12^ cm^–2^ (6-fold enhancement) then gradually decreases and finally saturates at ∼30% after ∼8 × 10^12^ cm^–2^. Earlier studies have shown that the modulation of  of neutral (*X*) and charged (*T*) states can be implemented with gate voltage,^18,19^ temperature,^19^ screening due to interlayer charge transfer,^20,21^ strain^22^ or magnetic field.^23^ Importantly, the modulation of the neutral states by electrostatic doping is limited to the low carrier density regime only. Hence, the dynamic modulation of  in charged states is of particular interest.

Since the *n* doping is increasing linearly with the gate voltage, the nonmonotonic behavior of  can be understood as a product of parallel contributions from different depolarization mechanisms. Here, the nonmonotonic behavior is explained using a combination of factors such as long-range electron–hole exchange (*J*^*LR*^) (the short-range exchange interaction is negligible due to 3-fold rotation symmetry of the monolayer,^13,20,47^*V*_*g*_-dependent dispersion of quinton state in exciton momentum (*q*) space, and the corresponding momentum scattering rate (γ_*p*_). Recently, the Maialle-Silva-Sham (MSS) mechanism caused by the intervalley electron–hole (*e*–*h*) exchange interaction has been suggested to dominate the spin and valley relaxation in monolayers.^17,20,47,59^ Here, the *J^LR^(q)* acts as a momentum-dependent effective magnetic field where different multiparticle energy species with a different center of mass (CoM) precess around the effective magnetic field with different frequencies.^14,20,21^ The exchange-driven steady-state valley polarization can be expressed as^19,59^
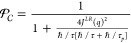
2Here τ refers to the radiative lifetime of the state, τ_*p*_ is the depolarization related to momentum scattering, with  = γ_*p*_ being the momentum scattering rate, while the intervalley exchange interaction with CoM momentum is *q*, and *J^LR^(q)* reads as^47,59^
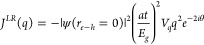
3where |ψ(*r*_*e*–*h*_ = 0)|^2^ is the overlap between electron and hole wave function which is approximated by , *a*_*B*_ refers to the Bohr radius, *a* is lattice constant, *t* is the hopping parameter, *E*_*g*_ is the electronic bandgap, and *V*_*q*_ is the Coulomb potential defined by , where *e* is the elementary charge, ϵ is the effective dielectric, and *r*_0_ is the fitting parameter.

For recombination of the neutral excitons, the radiative photon is confined within a light cone due to the conservation of momentum, resulting in a small kinetic energy. In contrast, the constraint on the recombination of charged states is not as stringent, as the energy of the final state for recombination of the charged state changes with *q*.^48,60^ Thus, a recombination process of charged states can also occur at different *q* values. While the recombination at *V*_*g*_ ≈ 0 V is from the band edge *q* = 0, the recombination occurs at higher *q* values when *V*_*g*_ increases. Now, the difference in effective mass between the five-particle *Q* state and the three-particle *T*_*D*_ state results in pronounced kinetic energy. To conserve energy and momentum, the emitted photon undergoes a redshift over the applied voltage range as shown in Figure 2d. The energy difference can be mapped from the following relation:
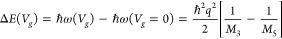
4Here *M*_3_(*M*_5_) is the mass of the three-(five-) particle species, and  denotes the photon energy for given *V*_*g*_. As the carrier density or *n* doping increases, the *J*^*LR*^(*q*) also increases due to *q*-space dispersion. The *V*_*g*_-dependent *J*^*LR*^(*q*) can be used in Equation 2 to match the experimental  value for a given τ. Further details on this are described in Supporting Information S4. Assuming the radiative lifetime is constant across *n*, we can further extract the momentum scattering rate (γ_*p*_).

In Figure 2e, γ_*p*_ gradually increases with carrier density at the low-doping regime and then saturates after *n* reaches ∼4 × 10^12^ cm^–2^. If the τ of the charged state is constant over the carrier density, we observe a strong variation in γ_*p*_ for the charged excitons, owing to their increased *q*-space dispersion. The initial increase in momentum scattering leads to the enhancement of valley polarization observed in Figure 2c. Similar behavior was recently reported for monolayer TMDs, where the spin/valley polarization of neutral exciton increases with an increase in carrier density,^14,19^ and this is attributed to the motional narrowing effect.^61,62^ The earlier studies quantitatively modeled the variation of the degree of circular polarization with scattering. It was experimentally shown that increasing scattering leads to a nontrivial enhancement in exciton polarization.^20,21^ However, with higher doping, this effect vanishes with enhanced kinetic energy and *J*^*LR*^(*q*) (see Supporting Information S4).^15^ This is also reflected in the width reduction of the PL peaks at the low-doping regime, as shown in the inset of Figure 2c and Supporting Information S3. Contrary to the almost constant, small value of the exchange interaction for the neutral exciton, the *J*^*LR*^(*q*) of the charged states show pronounced variation,^17,19,59^ with increasing carrier density as shown in Figure 2f. Also, the CoM momentum distribution of the *Q* state across the carrier density, extracted from the experimental red-shift of the peak position (from Figure 2b and Equation 4) presented in the inset of Figure 2f.

**Figure 3:**
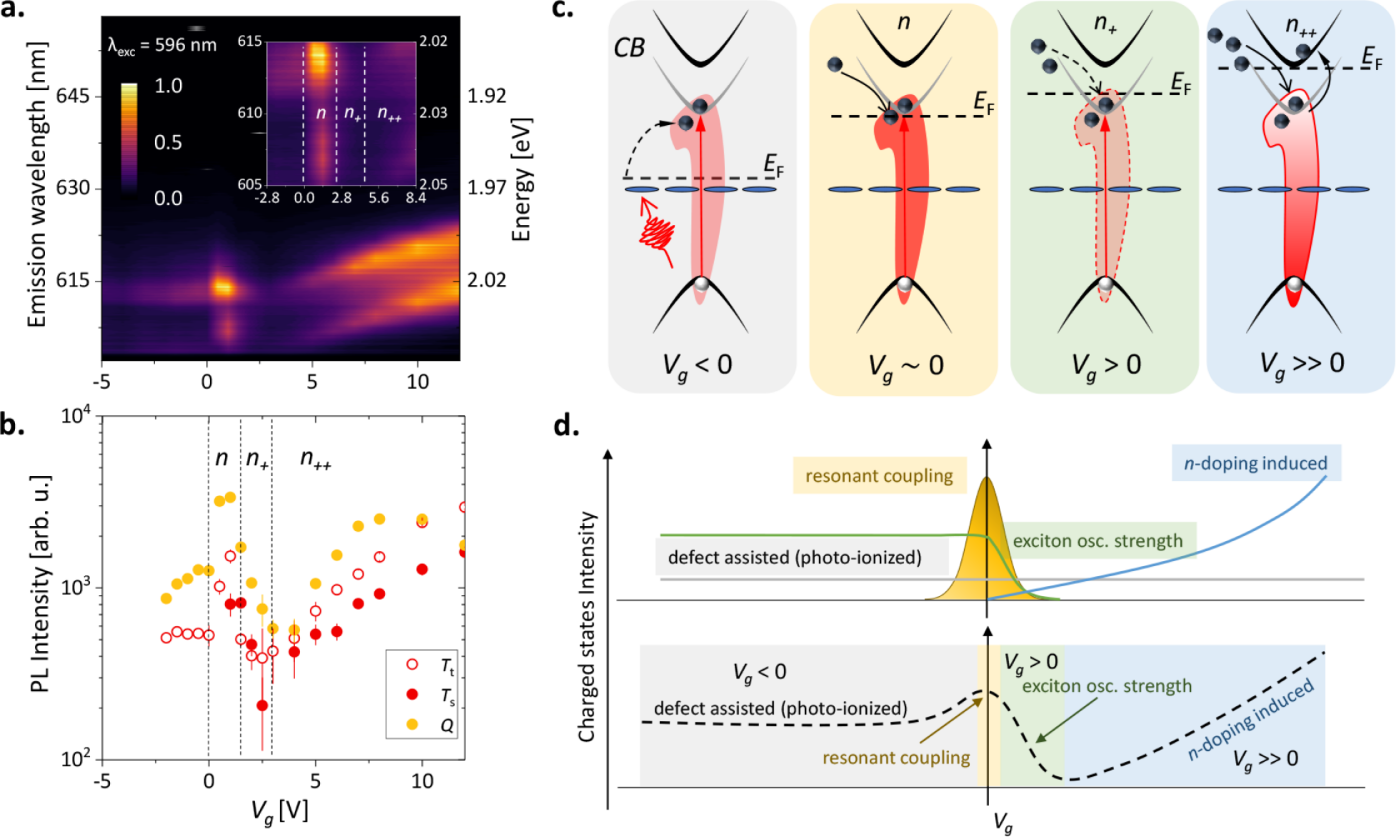
Modulation of resonantly coupled charged excitonic complexes under resonant excitation. (a,b) Gate-dependent intensity mapping of the charged states under resonant excitation with circularly polarized light in copolarized (*I*_*co*_ or σ^+^/σ^+^) mode. A zoomed-in picture is presented in the figure inset divided into four different regions. The intensity profile follows a nonmonotonic behavior. Nonmonotonic intensity modulation of the charged states corresponding to *T*_*s*_, *T*_*t*_ and *Q* states is presented in b. The vertical dashed lines demarcate four different regions same as in a. (c) Formation of the charged states are represented schematically for four different conditions (Three particle states are shown in the schematics for simplicity. The same argument holds for five particles also). For *V*_*g*_ < 0 V (gray background), the charged states are formed either through defect/impurity-assisted processes or through the photoionization effect where defect states are shown by the discontinuous blue band below the bandgap. This is a relatively weak and inefficient process, so the population of charged states is low. When *V*_*g*_ becomes slightly positive, the resonantly formed excitons (red vertical arrow) can efficiently capture the electrically injected (shown by the curved arrow) carriers and hence there is a strong population of charged states (*n*: yellow background). For the *n*_+_ region, the exciton oscillator strength decreases with increased *n*-doping. The net population density of the charged states is determined by the product of the exciton population (i.e., oscillator strength of the neutral exciton) and the coupling parameter under resonance. As a result, the density of the resonantly coupled charged states decreases in the *n*_+_ region, which is indicated by a green background. Finally, for *V*_*g*_ ≫ 0 V, the system becomes electron-rich and the *n*-doping induced charged state carrier population starts to grow as for the nonresonant excitation scenario (*n*_++_: sky background). The dashed black line denotes the corresponding Fermi levels (*E*_*F*_) at each doping density. (d) The upper panel schematically represents the individual expected intensity profile of the charged states as a function of *V*_*g*_ for the four cases described above while the combined effect is represented in the bottom panel as a reference describing the anomalous experimental trend-line (with black dashed line). The color coding refers to the different cases for four different regions of *V*_*g*_ described above.

As discussed earlier, the formation of the charged states under resonance can be controlled efficiently via resonant coupling between neutral and charged excitons.^25,27,63^ The neutral exciton population is more efficient with resonant excitation, while the charged states population can be modulated via external doping. Hence, by changing the carrier density at the exciton resonance creates a channel to control the coupling parameters between neutral and charged states. The 2D colorplot in Figure 3a represents the PL for the lower energy states relative to *X* across the range of *V*_*g*_ (see the Methods section for further details). Here, the excitation energy is set to neutral exciton resonance (596 nm). Unlike the nonresonant case, the intensity of the *T* and *Q* charged quasi-particles excited at the exciton resonance exhibit a nonmonotonic gate dependence, as shown in Figure 3b.

For *V*_g_ < 0 V, we do not observe evidence of significant external doping in Figure 3a (*p* doping is highly unlikely,^54,55^ and the Fermi level stays within the bandgap. Hence, the exciton population remains unaltered throughout this region (as shown in Figure 3a). Also, since the excitation is exactly at the excitation resonance, there is a scarcity of optically generated free carriers. Thus, the charged excitons are formed either through defect-assisted processes^64^ or via photoionized carriers.^65^ As a consequence, there is no significant variation in the population of charged excitons, and their total density remains relatively low.

At small positive gate voltages (0 < *V*_*g*_ < 1 V), we observe in Figure 3a,b that the population of free electrons starts to increase (denoted as *n*), promoting the formation of charged *T* and *Q* species. The population of charged excitons is further expected to be enhanced by the resonant condition that assists the down-conversion of the neutral excitons.^53^ The resonantly formed excitons can now efficiently capture carriers and form multiparticle states. In this regime, the resonant coupling between neutral and charged excitons reaches the maximum, resulting in an order-of-magnitude increase of the charged exciton densities. Under resonance, the net population density of the charged states is a product of the exciton population (oscillator strength of the neutral exciton) and the coupling parameter. As the doping increases with increasing *V*_*g*_, the exciton oscillator strength decreases, causing a decrease in the population of coupled charged excitons, reaching its minimum at *V*_*g*_ = +2.5 V (denoted as *n*_+_ in Figure 3a,b).

For *V*_*g*_ > 3 V, where the system is electron-rich in Figure 3a,b, the steady-state population of the charged states is not resonantly coupled. This can further be confirmed by performing the PLE experiment similar to Figure 1d but in an electron-rich environment (shown in Supporting Information S5). As a consequence, it grows with increasing doping, similar to nonresonant excitation, as shown in Figure 2a (denoted as *n*_++_ region).

Thus, the optical intensity of the charged states shows a distinct behavior under different carrier densities under positive bias under resonant excitation, especially with a strong presence of bright *T* states along with the *Q* state. The significance of this unusual relationship between optical intensity and gate voltage is evidence of the fact that the population of the charged states primarily depends on the neutral exciton population, especially for the low-doping regime. This anomalous behavior of the charged states population is a unique example of the doping dependence of the resonant coupling between neutral and charged states. The above plausible scenarios are schematically presented in Figure 3c for four different regimes (*V*_*g*_ < 0 V, and three *n*-doping regions marked as *n*, *n*_+_ and *n*_++_ are presented with different background colors while the black dashed line represents the Fermi energy at different doping levels). Resonant excitation persists throughout the applied voltage and is shown via a red vertical arrow, and the relatively weaker processes are denoted by the dashed curved arrows. The intensity profile for the individual effects is demonstrated in the top panel of Figure 3d, and the collective trend is depicted in the bottom panel of the same figure (the color coding follows the same conventions as those in the prior figure). The intensity modulation of charged states with the change in gate voltage is due to the combination of the various processes described above.

Similar to the nonresonant regime, the motional narrowing effect for valley polarization of charged excitons persists with resonant excitation of neutral species at low doping densities (<4 × 10^12^ cm^–2^) (see discussions in Supporting Information S6 and S7). The efficient down-conversion from the neutral to the charged states can be enhanced in the presence of resonant optical phonons (Fröhlich interaction).^66^ Indeed, the energy difference between the *X* and *T* states matches quite well with the vibrational phonon mode ( mode) of the monolayer WS_2_. Hence, the existence of phonon-driven “virtual trion” is highly probable.^67^ However, it has been reported that only optical phonons are favorable for this efficient down-conversion, and so the same argument does not hold for the efficient generation of the *Q* state from the *X* state resonance.^67,68^ While there is no optical phonon with energy that can match the energy difference between *Q* and *X* excitons, the down-conversion may result from interactions with overtones or a combination of (optical and acoustic) phonon modes^69^ (see Supporting Information S9 for further details).

**Figure 4:**
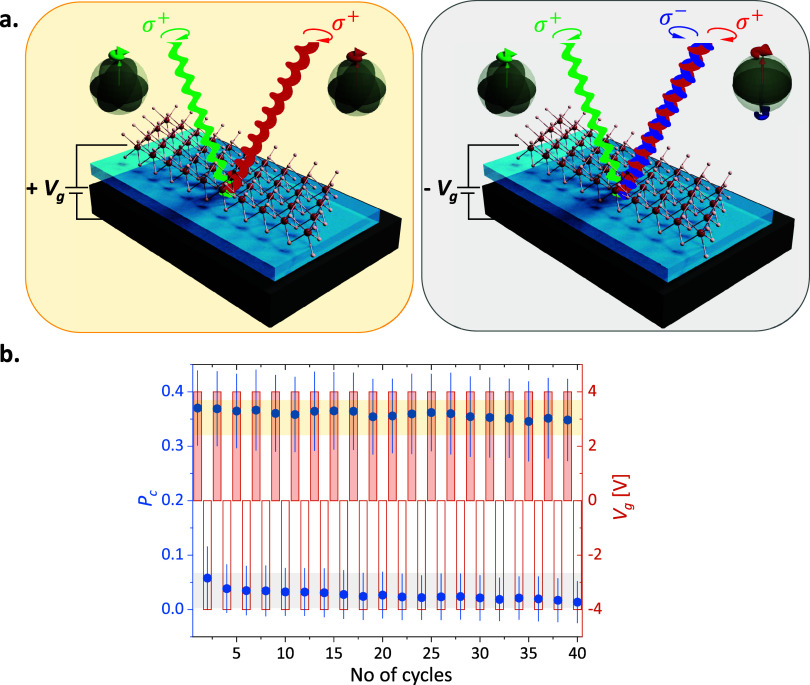
Electrical switching of polarization for quinton states. (a) The degree of circular polarization corresponding to the quinton states can be electrically switched from a high-polarized state to a low-polarized state by altering the polarity of *V*_*g*_. The circular polarization retains for the bright *Q* states when *V*_*g*_ is positive i.e., the system is electron-doped as shown in the left panel. However, the valley information is lost when the polarity of the *V*_*g*_ is altered as shown in the right panel. In our experimental scheme, we excite with σ^+^, and detect σ^+^ photon (shown in red) from the *Q* state with a positive voltage, while getting the equal intensity of both the helicity of photons with negative *V*_*g*_ (shown in red and blue). (b) The device operation is demonstrated over several cycles by sequentially altering the polarity of *V*_*g*_ and recording the corresponding *P*_*c*_ over a wavelength window of 618–624 nm. Here, the *V*_*g*_ is switched between ±4 V and the *P*_*c*_ is switched between high (yellow-shaded region) and low-polarized states (gray-shaded region) efficiently without any noticeable variations. The error comes from the statistical average within the specified wavelength range.

In the final part of the manuscript, we discuss a proof-of-concept device that demonstrates a valley polarization switch with reproducible performance over multiple gate cycles. This device (denoted as D2) is developed based on the core findings from the earlier characterized monolayer WS_2_ device (denoted as D1). Both D1 and D2 share identical device architecture and active material, as further detailed in the characterization of the D2 sample (sample characterization is presented in Supporting Information S10). Conceptually, the proof-of-concept device leverages the strong gate-dependent behavior of the five-particle *Q* states, initially described for D1 in Figure 2c. The consistent switching behavior of *Q* states in both devices highlights the robustness of the observed phenomena. By altering the *V*_*g*_ polarity, the *P*_*c*_ can be switched from the high polarized state to a low polarized state. This is schematically presented in Figure 4a. In the case of D2, electrical switching of the polarization corresponding to the *Q* state was observed over extended switching cycles, as shown in Figure 4b (the switching behavior of polarized states in D1 is detailed in Supporting Information S11). The consistent switching behavior of *Q* states in both devices highlights the robustness of the observed phenomena.

To demonstrate stable switching control, we set a threshold of <10% for the low state and >40% for the high state, for both nonresonant and resonant excitation. For D2, we have performed 40 cycles of sequential switching of the *V*_*g*_ between +4 V to −4 V. At + 4 V, the carrier density in the monolayer increases due to *n* doping, favoring the formation of charged states with high optical intensity, resulting in a high polarization contrast, primarily from the *Q* state. Here, we have recorded the *P*_*c*_ within the wavelength window of the *Q* state and presented the statistical mean value of the *P*_*c*_ along with the standard deviation. A similar procedure was followed when *V*_*g*_ = −4 V. The population of the *Q* state is low for *V*_*g*_ < 0 V, leading to low *P*_*c*_. Consequently, the shift in *V*_*g*_ from negative to positive causes the response from the *Q* state to shift from high *P*_*c*_ to low *P*_*c*_. The recorded *P*_*c*_ of device D2 shows stable switching in either high or low-polarized states within measurement errors. The electrical switching of valley polarization corresponding to the *Q* states is represented in Figure 4b in the main text with quasi-resonant excitation, while the same for the first device is shown in Supporting Information S11.

## Conclusion

In conclusion, our work demonstrates the ability to deterministically control the valley-polarized emission from charged exciton states in monolayer TMDs using an external electrical bias, thereby overcoming the limitations of neutral excitons. Through careful selection of scattering mechanisms, we can generate strong valley-polarized emission of a given chirality or suppress it on demand. We also found that this polarization is resilient against environmental noise fluctuations associated with our devices. By using electrical bias, we can efficiently vary the exchange interaction and scattering rates of charged states, accentuating the motional narrowing effect, yielding a deterministic valley-polarized emission from charged states and simultaneously boasting optical brightness. Specifically, we show electrical control over charged biexciton (quinton) which thereby enabled the deterministic control of valley polarization in monolayer WS_2_ devices where *P*_*c*_ can be tuned from <10% up to >60%, representing a 6-fold increase under electrical bias. While it is well-known that the population of the charged states can be electrically controlled, we want to emphasize reproducible and stable electrical switching of the valley polarization in monolayer using charged biexciton with brighter optical intensity. This deterministic switching inspires the possibility of manipulating valley quantum states using charged excitons. The exquisite electrical tunability of the spectral resonance of charged states promises resonant control of valley polarization through the intricate interplay between the charged and neutral exciton states, and the electrical control of resonant valley polarization bodes well for exploiting the valley degree of freedom for information storage and manipulation. This research represents a key step toward advanced optoelectronic devices, enabling possibilities for applications such as facilitating the realization of highly nonlinear chiral emissions in microcavity systems,^70^ valley-selective optical switching and valley-based quantum technologies with enhanced stability and reliability.

## Methods

### Device Fabrication

The prototype device was fabricated by sequential transfer of individual layers identified by optical microscope via dry transfer method using micro manipulators. The layers were exfoliated from commercially available flux-grown materials (WS_2_ from 2D semiconductors, and graphene from HQ Graphene) on the top of polydimethylsiloxane (PDMS) using Nitto tape and transferred on prepatterned contacts over a SiO_2_(285 nm)/Si substrate. The contacts were defined through e-beam lithography using PMMA resist. The contact pads were made with 5/40 nm Cr/Au deposited by thermal evaporation followed by liftoff in Microposit remover 1165 solution. The monolayer is connected to the metal pad using a graphite layer. The entire stack was annealed inside a vacuum chamber (10^–6^ mbar) at 250 ^◦^C for 6 h for better adhesion between the layers.

### Measurement Setup

#### Polarized μPL

For low-temperature circular dichroic photoluminescence (CDPL), the sample was placed inside a cryostat on top of a motorized stage. The excitation source for all-optical spectroscopy experiments was an NKT supercontinuum source (repetition rate 20 MHz, pulse duration 80 ps for 5 nm bandwidth), filtered through a 770 nm short-pass filter and linearly polarized with a polarizing cube beam splitter. To create nonresonant excitation at 570 nm, the white supercontinuum was subsequently filtered through a 6 nm bandpass filter centered about 568 nm. The filtered beam was then circularly polarized through an achromatic λ/4 retarder and focused onto the sample through a ×50, NA = 0.6 microscope objective lens. The spot size on the sample is about 3 μm in diameter.

Sample photoluminescence was captured through the same objective lens and converted from circular to linear polarization basis through the same achromatic λ/4 retarder. Excitation light was removed by passing the emission beam through a long pass filter. The emission beam was then split into two orthogonally polarized beams using a polarizing beam splitter and separately focused into two multimode fibers. The fiber outputs were then dispersed by an Acton SP300 spectrograph and simultaneously detected on nonoverlapping regions of a Princeton Instruments EMCCD camera.

#### PLE Measurements

In resonant excitation and PLE experiments, the white supercontinuum beam was passed through a home-built prism monochromator, where the light was dispersed by an equilateral glass prism and then focused by a lens onto a pinhole. The focusing lens was mounted on a motorized stage to select wavelengths in the experimental range (580–600 nm). The selected wavelength had a bandwidth of ∼1 nm and a maximum power of ∼10 μW. For PLE, the emitted photons are passed through a 600 nm long-pass filter before sending it to the spectrometer.

#### Gate Dependent Spectroscopy

For gate-dependent spectroscopy, we have used Keithley 2450 SMU as a source meter. The sample placed inside the cryostat was mounted to the chip carrier with wire bonding and connection to the SMU. The gate leakage current was below tens of pA for the different sets of measurements. For gate-dependent resonant excitation spectroscopy with 596 nm, the color line selected from the PLE path was passed through another 600/10 nm bandpass filter. We have estimated the carrier density from the following relation as *nq* = *CV*_*g*_ where *n* is the carrier density, *q* is the elementary charge, *C* is the effective capacitance and *V*_*g*_ is the applied gate voltage. *C* is calculated further with the relation as *C* = [ϵ_*r*_ϵ_0_/*d*]|*V*_*g*_|. Here, ϵ_0_ = 8.85 × 10^–12^ F·m^–1^ is the permittivity of free space, ϵ_*r*_ = 3.9^70,71^ is the *h*BN dielectric constant and *d* = 40 nm is the total thickness of the *h*BN layers.

## Associated Content

A preprint version of this manuscript is available online.^72,73^

## Supporting Information Available

Supporting Information from S1 to S11 with the following details as PL characterization of device D1; peak fitting for nonresonant excitation in D1; gate dependent PL with nonresonant excitation; exchange interaction dependent modulation of valley polarization; PLE of device D1 and D2; gate dependent PL for resonant excitation; sample peak fittings for resonant excitation; spatial microscopy of the device; exciton phonon interaction under resonant condition; gate dependent polarization switching for D2; switching of valley polarization for D1 (PDF)
