# Supplementary material for: Electrical Control of Valley Polarized Charged Exciton Species in Monolayer WS2
Source: ACS Nano. 2024 Oct 22;18(44):30805–15. doi: 10.1021/acsnano.4c11080 (PMC11544929; doi:10.1021/acsnano.4c11080)
Supplement: Supplementary file 1 — nn4c11080_si_001.pdf [file nn4c11080_si_001.pdf]

Supporting Information: Electrical control of valley polarized charged exciton species in monolayer WS_2

Sarthak Das,[*,†,‡] Ding Huang,[†,‡] Ivan A. Verzhbitskiy,[†,‡] Zi-En Ooi,[†] Chit Siong Lau,[†,‡,¶] Rainer Lee,[†,‡] Calvin Pei Yu Wong,[†] and Kuan Eng Johnson Goh[*,†,‡,§,‖]

†*Institute of Materials Research and Engineering (IMRE), Agency for Science, Technology and Research (A*STAR), 2 Fusionopolis Way, Innovis #08-03, Singapore 138634, Republic of Singapore*

‡*Quantum Innovation Centre (Q.InC), Agency for Science Technology and Research (A*STAR), 2 Fusionopolis Way, Innovis #08-03, Singapore 138634, Republic of Singapore*

¶*Science, Mathematics and Technology, Singapore University of Technology and Design, 8 Somapah Road, 487372, Singapore*

§*Department of Physics, National University of Singapore, 2 Science Drive 3, Singapore 117551, Singapore*

‖*Division of Physics and Applied Physics, School of Physical and Mathematical Sciences, Nanyang Technological University, 50 Nanyang Avenue, Singapore 639798, Singapore*

E-mail: ds.sarthak.92@gmail.com; kejgoh@yahoo.com

Supporting Information S1

The figure below shows the optical image of the device fabricated using the dry transfer technique. We have completed the multi-peak fittings to analyze the power-law for each energy state. The energy peaks of the individual states remain nearly consistent regardless of the optical power applied. The exciton (X), trion (T) and charged biexciton (Q) peaks are extracted from Lorentzian multi-peak fittings. The corresponding power law (where intensity, $I \propto P^{\alpha}$ with P is the excitation power and α is the exponent of fittings) is presented in the main text. The representative Lorentzian multi-peak fittings are done to extract the energy of the individual peaks. The binding energy of the Q peak is $\sim$ 57 meV. Additionally, the circular polarization resolved PL with the degree of circular polarization of the same spectra (with 570 nm ps pulsed excitation) is represented separately.

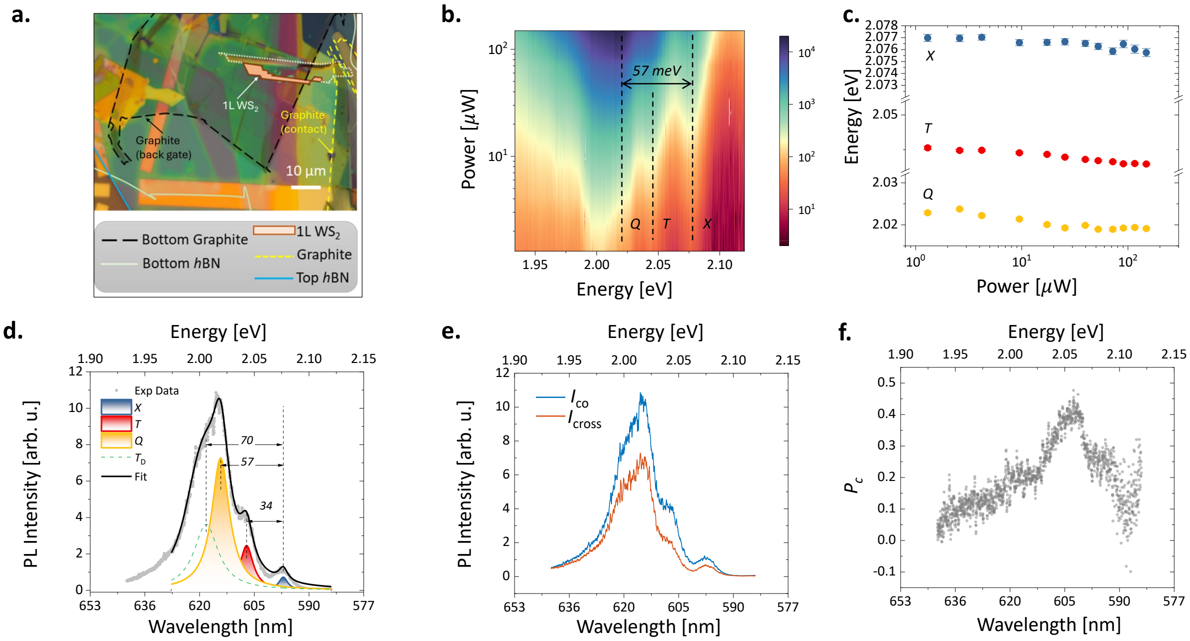

Figure 1: **PL characterization of the device D1.** (a) Optical image of the device under test fabricated using the dry transfer method. Individual layers are outlined for reference. The monolayer region is shaded and connected to a metal pad using a graphite layer. (b) The colour plot of the PL evolution for the power-dependent PL spectra from D1 under 570 nm ps pulsed excitation, where dashed vertical lines represent the peak positions of different energy states. The binding energy of the Q state is $\sim$ 57 meV across the power range. (c) The peak position of the energy states extracted across the input power shows minimal variation throughout the power range. (d) Sample multi-Lorentzian peak fitting of the PL spectra showing the primary contribution from the three dominant energy states analyzed as exciton (X), trion (T) and quinton (Q) with excitation power $=$ 70 μW. (e) Circular polarization resolved PL spectra from the sample in (e) and the corresponding degree of polarization (P_c) in (f).

Supporting Information S2

Representative spectra from the 2D map of gate-dependent photoluminescence (PL) and P_c modulation (as shown in Figure 2a of the manuscript) are displayed below.

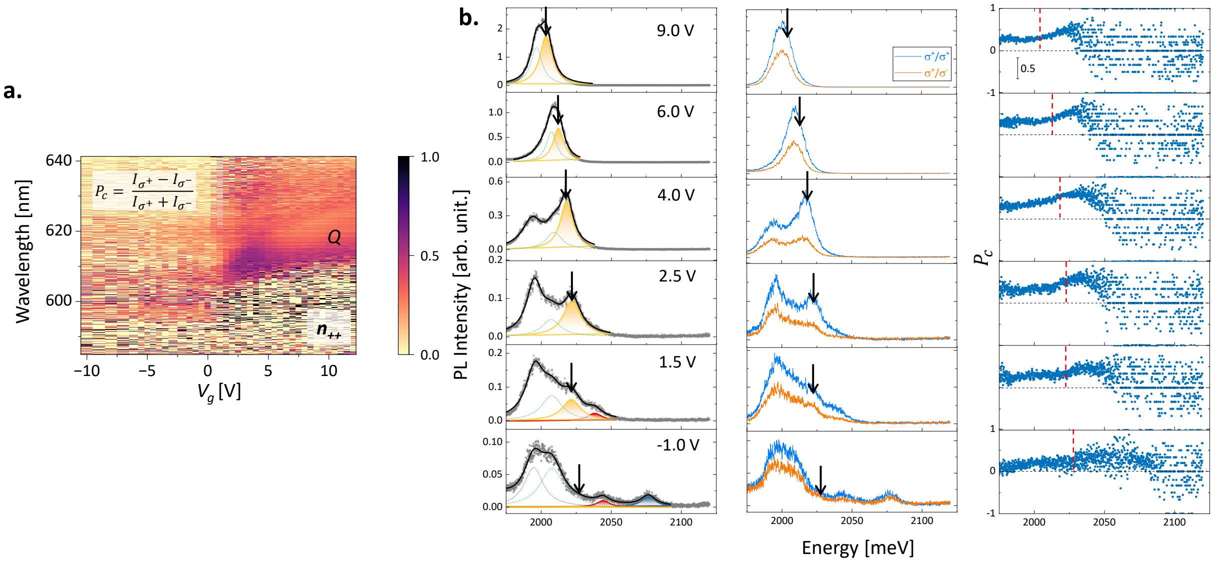

Figure 2: **Sample peak fittings for non-resonant excitation at λ_{exc} = 570 nm for D1.** (a) Gate-dependent 2D map of the P_c across the wavelength range. The P_c contrast is the maximum for the Q state. (b) The left panel shows bias-dependent representative PL spectra from D1 for different gate voltages (presented in the main text for pulsed 570 nm non-resonant excitation 2D map), while the middle panel presents the corresponding polarization-resolved PL spectra. The Q energy state is highlighted with a yellow-shaded region for reference, showing the intensity modulation and spectral shift. Under high carrier density, the spectra are dominated by the Q state. The individual spectra for given V_g under σ^+/σ^+ (σ^+/σ^-) excitation in blue (orange) is presented in the middle panel. The right panel shows a vertical line cut from the P_c map at different V_g from Fig. 2a.

Supporting Information S3

The gate-dependent color plot (in linear scale) for the non-resonant excitation (λ_{exc} = 570 nm) under cross-polarized mode recorded $in-situ$ with the co-polarized mode, is presented below. The maps demonstrate a clear evolution of the PL spectra, where the primary contribution comes from the quinton states.

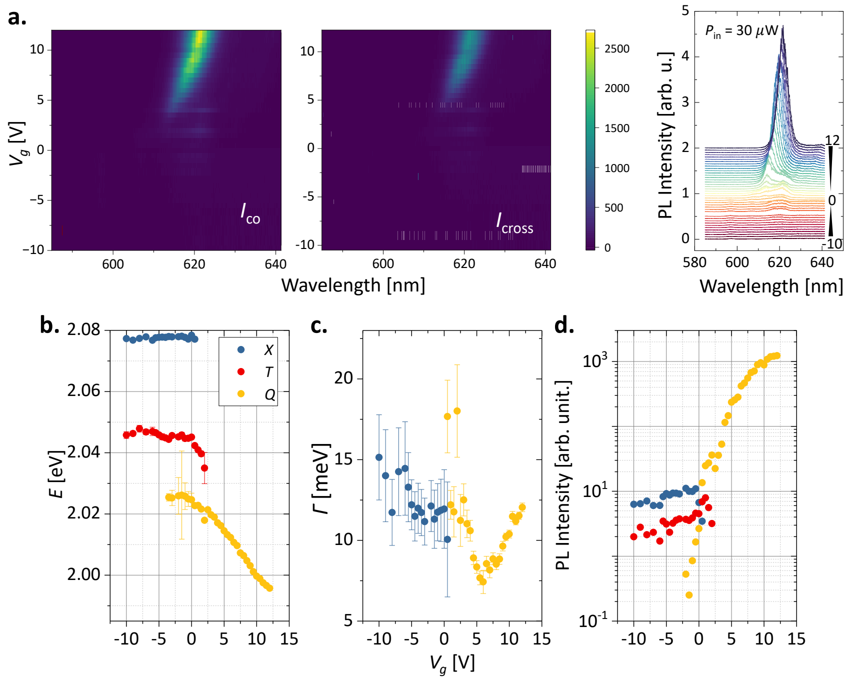

Figure 3: **Gate dependent modulation of μPL with non-resonant excitation.** (a) The two-dimensional color plot of the gate-dependent PL map in linear scale for co- and cross-polarized modes (Colorbar is kept at the same scale for reference). The map shows a clear evolution of the quinton states with increasing carrier density. The individual spectra for co-polarized mode are presented in the right panel across the V_g. (b) The spectral position of the energy states extracted from Fig. 5a (cross-polarized) as a function of V_g. (c) The modulation of FWHM of the Q state with V_g while the FWHM for the X state remains unaltered for $V_g < 0$ V. (d) The intensity modulation of the energy states over the applied V_g extracted from 5a (cross-polarized). The charged states are more responsive towards external bias and the brightness increases as the n-doping increases.

Supporting Information S4

Equations (2) - (4) from the main text provide the exchange interaction of the charged states over the q-space. The valley polarization ($\mathcal{P}_\mathcal{C}$) decreases as it moves away from the zone corners. Additionally, $\mathcal{P}_\mathcal{C}$ also depends on the ratio, r, of the radiative lifetime of the state to its scattering rate. To illustrate this, the $\mathcal{P}_\mathcal{C}$ of the Q state for non-resonant excitation from the experimentally mapped q-space (from figure 2a-b) with changing carrier density is plotted in relation to the radiative lifetime.

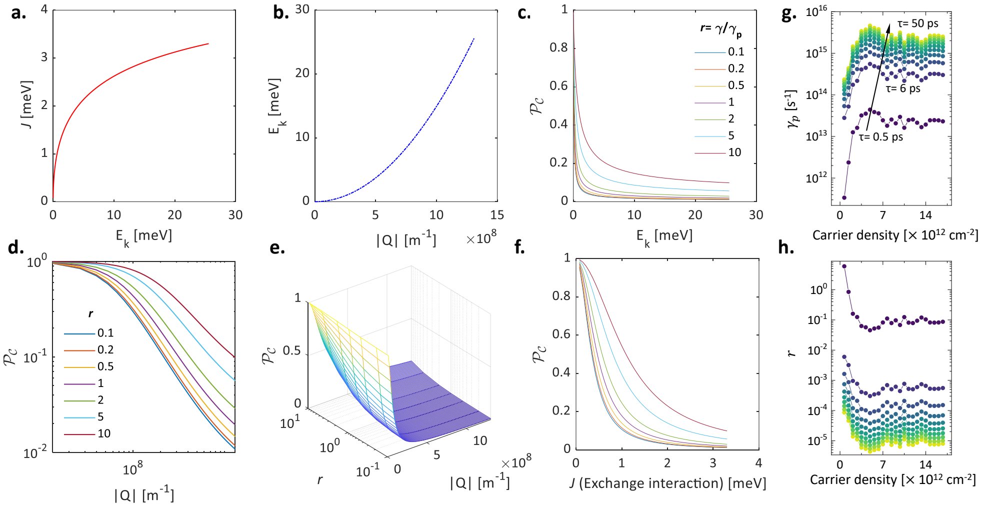

Figure 4: **Exchange interaction ($J^{LR}_{e-h}(q)$) dependent modulation of valley polarization($\mathcal{P}_\mathcal{C}$) of the charged states.** (a) Plot of exchange interaction[1,2] variation as a function of increasing kinetic energy (E_k). The E_k is relatively small for the excitons as their radiative recombination is limited within the light cone. Hence, the $J^{LR}(q)$ is small and approximated as constant for neutral states, which is a stark difference compared to the charged states.[3] (b) The E_k dispersion for the five particle states in q-space. The range of E_k is limited to match the experimental redshift. (c-d) With increasing E_k the $\mathcal{P}_\mathcal{C}$ decreases as the q-space dispersion increases (plotted in Fig. 4d). However, the valley polarization is also dependent upon the ratio (r) between the decay rate (γ) and the scattering rate (γ_p). (e-f) The overall picture of the $\mathcal{P}_\mathcal{C}$ modulation as a function of r and q-dispersion (in Fig. 4e) as well as with the exchange interaction in Fig. 4f. (g) The extracted scattering rate γ_p from the experimental $\mathcal{P}_\mathcal{C}$ value across the carrier density for varying radiative recombination time τ. The variation in γ_p across the carrier density with τ = 50 ps is presented in the main text. (h) The decay rate normalized by the momentum scattering rate, r, ($r = \frac{\gamma}{\gamma_p}$) against the carrier density for varying τ. Note that if the τ changes with V_g (or carrier density), the r can be adjusted accordingly to match the experimental $\mathcal{P}_\mathcal{C}$.

Supporting Information S5

The PLE is performed when the monolayer is electron-doped in order to demonstrate the disappearance of the resonant coupling between neutral and charged states. The prominent dependence of the charged states exactly at excitonic resonance is missing at $V_g = +4$ V as presented in the upper panel of the figure below. Similarly, We have repeated the PLE experiment on a separate sample (D2) by changing the wavelength in a resolution of ~ 0.3 nm (by controlling the motorized stage) where we can observe a similar trend where charged

exciton PL intensity grows as we approach towards the neutral exciton energy resonance (in bottom panel). However, the exact evolution could not be observed as the neutral exciton resonance occurred beyond our detection range.

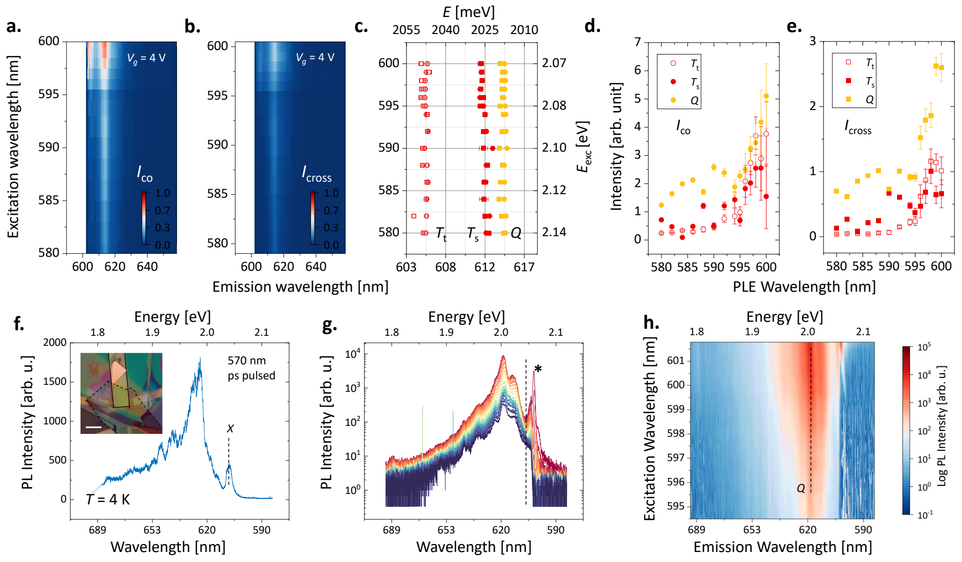

Figure 5: **PLE characterization of the device D1 and D2.** (a-b) Colorplot of excitation wavelength-dependent circularly polarized emission for the charged states in an electron-rich environment with a constant $V_g = 4$ V. The charged states are not resonantly coupled with the neutral states contrary to the figure presented in the main text. (c) Wavelength-dependent energy positions of the charged states. There both the intra (T_s) and inter valley (T_t) trions are visible in the n-doped region. The circle (square) symbols denote the co (cross) polarized mode extracted from the fittings. (d-e) PLE wavelength-dependent intensity of the individual states shows interruption of the resonant coupling between neutral and charged states for both polarizations as there is no prominent signature at exciton resonance. (f) Low-temperature PL of sample D3 with 570 nm ps pulsed laser at $T = 4.2$ K. The exciton (X) is at 605 nm for this sample. Inset shows the optical image of the device with a monolayer region highlighted with a false colour representation. (g) Excitation-dependent (594-600 nm with a resolution of ~ 0.3 nm) PL spectra for D3. The asterisk (*) denotes the reflection from the laser line and the vertical dashed line represents the edge of the 600 nm long pass filter. The PL intensity monotonically increases with the long wavelength excitations and reaches the maximum as excitation approaches exciton resonance. (h) 2D colormap of the PL evolution of the charged biexciton where the vertical dashed line follows the intensity modulation corresponding to the Q state.

Supporting Information S6

The gate-dependent color plot for the resonant excitation ($\lambda_{exc} = 596$ nm) under cross-polarized (σ^+/σ^-) mode recorded $in-situ$ with the co-polarized mode, is presented below. The energy states behave similarly to the co-polarized mode along with a strong modulation in valley polarization similar to non-resonant excitation.

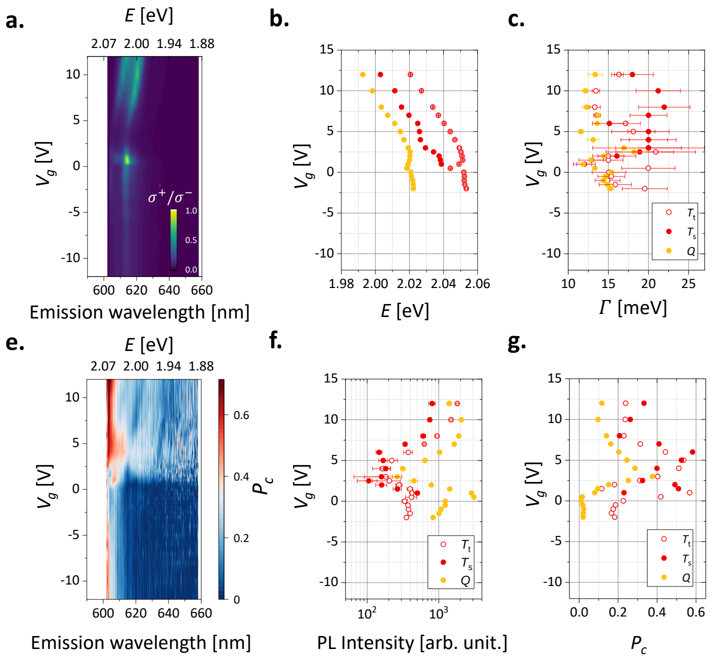

Figure 6: **Gate dependent spectroscopy for resonant excitation.** (a) 2D colorplot of the V_g dependent PL for $\lambda_{exc} = 596$ nm recorded for σ^+/σ^- emission mode. (b-c) V_g dependent energy positions and corresponding line-width (Γ) for the charged energy states for σ^+/σ^- emission mode. (e) The calculated colormap for V_g dependent degree of circular polarization for resonant excitation. While it is almost featureless for $V_g < 0$ V, the valley polarization shows a similar modulation to non-resonant excitation described in the main text for $V_g > 0$ V. (f-g) The anomalous modulation of the brightness of the charged states across the applied V_g while the modulation of the valley polarization is presented in Fig. 6g.

Supporting Information S7

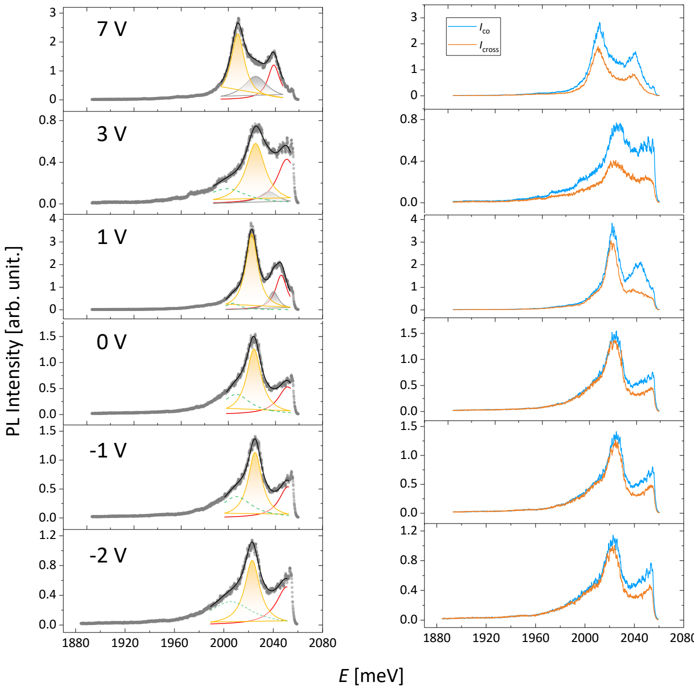

Figure 7: **Sample peak fittings for resonant excitation, λ_{exc} = 596 nm.** Sample peak fittings for different gate voltages (discussed in main text for 596 nm resonant excitation) where the Q state is highlighted in yellow for reference showing the intensity modulation and spectral shift in left panel. The individual spectra for given V_g under σ^+/σ^+ (σ^+/σ^-) excitation in blue (orange) is presented in the right panel.

Supporting Information S8

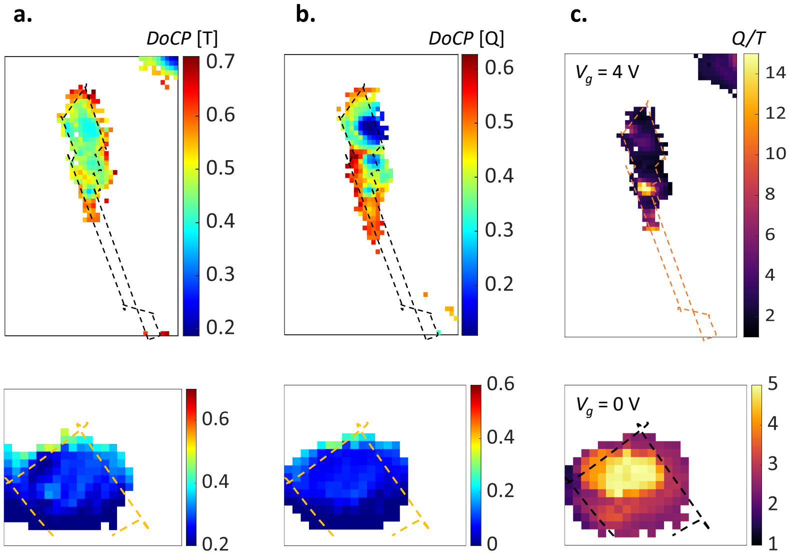

Figure 8: **Spatial microscopy of the device for resonant excitation.** (a-b) The top panel shows the spatial microscopy of the device under test in an electron-rich environment (with a constant $V_g = 4$ V) showing the valley polarization within 606 - 610 nm region (corresponding to the three particle negatively charged excitons or trions) in a and the same for five-particle negatively charged biexciton (within 610 - 614 nm) in b. The monolayer region is outlined for reference. The signal-to-noise ratio is kept $\sim$ 10 to remove the unwanted points. The bottom panel is for $V_g = 0$ V for the same excitation. (c) The spatial distribution of the intensity ratio between Q and T states showing the partial spatial inhomogeneity from the sample under test conditions. Thus, the gate-dependent μPL also has some dissimilar appearance over different locations within the same monolayer. The top panel is for $V_g = 4$ V bottom panel is for $V_g = 0$ V.

Supporting Information S9

The gate-dependent PL spectroscopy is presented against the energy difference with respect to the laser excitation ($\lambda_{exc} = 596$ nm) to correlate with the individual energy states with the available phonon modes.

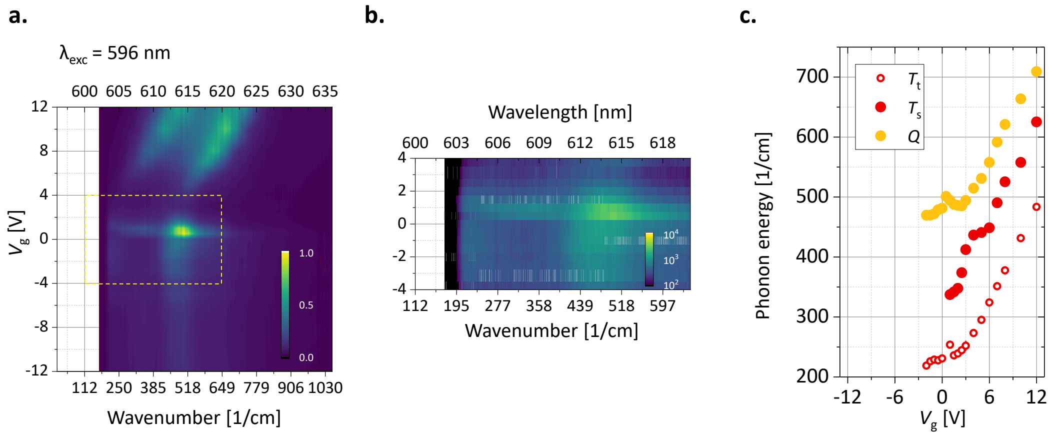

Figure 9: **Exciton phonon interaction under resonant condition.** (a) The 2D colormap presented in the main text relative to the phonon-energy difference from the resonant excitation. (b) Zoomed in view of the outlined area showing that the brightness intensifies due to efficient down-conversion from the neutral state when the trion state matches the optical phonon energy at $V_g = 1$ V.[4] (c) The shift of phonon energy difference from the excitation for different states over the applied V_g. While the energy difference of the three-particle state matches with available phonon modes,[4,5] the Q state does not directly correlate to any of the phonon modes for monolayer WS_2.

Supporting Information S10

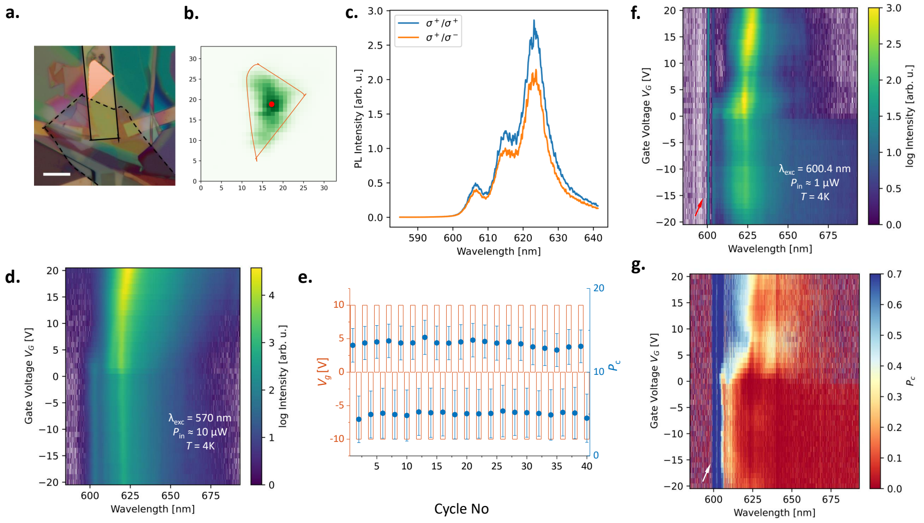

Figure 10: **Gate-dependent spectroscopy and polarization switching for D2.** (a) The optical image of the device under test in the upper panel, where the monolayer portion is highlighted with a false-color. The back metal gate boundary is marked with a black line, while the contact graphite is shown with a dashed-black boundary. The scale bar is 10 μm. (b) The two-dimensional PL mapping (within 16.5 μm × 16.5 μm area) of the luminescence intensity from the device within the 585–645 nm wavelength range at $T = 4$ K in co-polarized mode. The monolayer boundary is highlighted for reference. (c) The representative PL spectra for co- and cross-polarized mode from the map (the red dot) under 570 nm excitation with <10 μW excitation power (P_{in}), showing dominant features corresponding to different energy states. (d) Gate-dependent PL spectroscopy for non-resonant ($\lambda_{exc} = 570$ nm) excitation. The energy states produce similar observations discussed in the main text. However, due to the thicker dielectric, the carrier density is lower compared to the main text device. Hence, the spectral red shift corresponding to the dominant Q state is subtle for D3 compared to D1. (e) Switching between high- and low-polarized states with altering V_g for pulsed excitation with $\lambda_{exc} = 570$ nm. As previously discussed, the modulation in P_c is less pronounced in this case, and the applied voltage V_g must be increased to ±10 V to achieve a noticeable switch in P_c at a specific carrier density. (f-g) 2D colorplot of the gate-dependent PL and P_c for quasi-resonant ($\lambda_{exc} = 600.4$ nm) excitation. The P_c modulation here is remarkably higher, along with the anomalous intensity profile for the charged states against the carrier density. The arrows denote the laser line.

To validate our experimental results discussed in the main text, we have performed gate-dependent PL spectroscopy on device D3 with a similar architecture. Here, the back gate is replaced by a metal gate (Cr/Au) in place of a graphite gate, as shown in figure 10a. The device shows similar performance as the previous device (D1) discussed in the main

text. The exciton resonance ($\sim$ 605 nm) here is red-shifted compared to D1, likely due to different hBN thicknesses (in top and bottom) and the change in the binding energy.[6] Hence, for non-resonant excitation (λ_{exc} = 570 nm), the modulation in P_c is lower, likely due to excitation even further from the optical bandgap as shown in figure 10d-e. However, in quasi-resonant excitation (λ_{exc} = 600.4 nm), the gate dependence of the PL emission replicates the non-monotonic evolution of the charge states under electron doping, as shown in figure 10f. Moreover, the P_c can be modulated electrically (shown in figure 10g), as discussed for the device in the main text.

Supporting Information S11

By altering the polarity of V_g, valley polarization can be achieved in either the on or off state by considering a threshold. By applying a sequential series of opposite-polarity pulses of voltage V_g, the degree of circular polarization (P_c) of the charged states can also be altered. This is applicable for both the non-resonant and resonant excitations as shown below. The average P_c has been considered within a fixed wavelength window, ignoring the relative of the individual energy states over the applied $V_g > 0$ V (where the states do not show any spectral shift for $V_g < 0$ V).

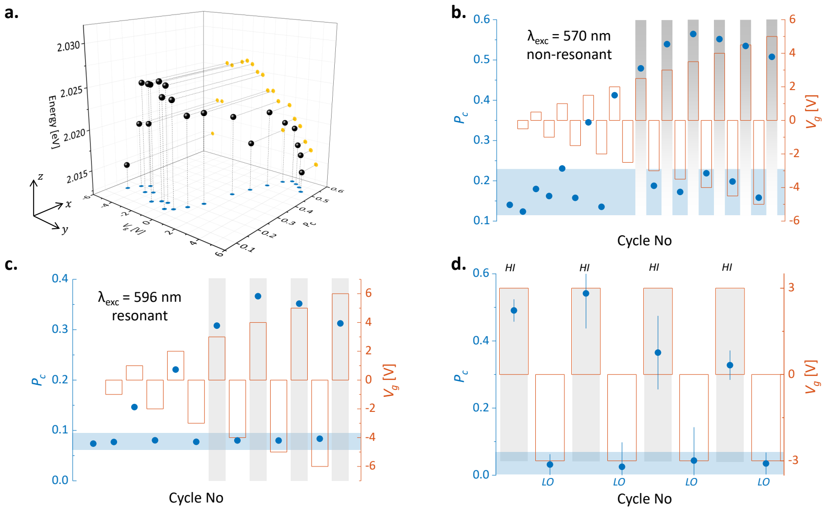

Figure 11: **Switching of valley polarization for D1.** (a) The 3D plot of the Q state under V_g variation (in y-axis) against its energy position (in z-axis) and P_c (in x-axis) from figure 2b-c from the main text under non-resonant excitation ($\lambda_{exc} = 570$ nm). (b) When an increasing V_g of alternating polarity (-6 V $< V_g <$ $+6$ V) is applied, the corresponding valley polarization also shows an alternating trend for the Q state. Considering the P_c within $0.1 - 0.2$ as low-polarized state (shown in the blue-shaded region) and $P_c > 0.4$ as high-polarized state for a wavelength window of 610-617 nm, the P_c can be realized as a voltage-controlled valley switch for non-resonant excitation. (c) The switching of valley polarization for resonant excitation. Here, the threshold can be considered as $P_c < 0.1$ for $V_g < 0$ V. The P_c from the individual states are slightly different as extracted from fitting. Here, the average P_c has been considered within a fixed wavelength window (609-616 nm) with a primary contribution from the Q state. (d) When a fixed V_g is applied with an alternative polarity, then also the P_c can be switched repeatedly between a high (HI) and a low (LO)-polarized state. Here, it is shown for $V_g = +3$ V and -3 V, with a fixed smaller wavelength window (614-616 nm). This can further be implemented for any voltage between $2 < |V_g| < 5$ V. This shows further deterministic control over photon energy along with voltage-controlled polarization tuning.

References

(1) Chen, S.-Y.; Goldstein, T.; Tong, J.; Taniguchi, T.; Watanabe, K.; Yan, J. Superior valley polarization and coherence of 2s excitons in monolayer WSe_2. *Physical review letters* **2018**, *120*, 046402.

(2) Wu, Y.-C.; Taniguchi, T.; Watanabe, K.; Yan, J. Enhancement of exciton valley polarization in monolayer MoS_2 induced by scattering. *Physical Review B* **2021**, *104*, L121408.

(3) Wang, H.; Zhang, C.; Chan, W.; Manolatou, C.; Tiwari, S.; Rana, F. Radiative lifetimes of excitons and trions in monolayers of the metal dichalcogenide MoS_2. *Physical Review B* **2016**, *93*, 045407.

(4) Van Tuan, D.; Jones, A. M.; Yang, M.; Xu, X.; Dery, H. Virtual trions in the photoluminescence of monolayer transition-metal dichalcogenides. *Physical Review Letters* **2019**, *122*, 217401.

(5) Molas, M. R.; Nogajewski, K.; Potemski, M.; Babiński, A. Raman scattering excitation spectroscopy of monolayer WS_2. *Scientific reports* **2017**, *7*, 5036.

(6) Gerber, I. C.; Marie, X. Dependence of band structure and exciton properties of encapsulated WSe_2 monolayers on the hBN-layer thickness. *Physical Review B* **2018**, *98*, 245126.
